# Potency of *Origanum vulgare* and *Andrographis paniculata* extracts on growth performance in poultry

**DOI:** 10.1016/j.vas.2022.100274

**Published:** 2022-11-30

**Authors:** Elvina J. Jahja, Riana Yuliana, Welinda Turianna Simanjuntak, Noer Fitriya, Anita Rahmawati, Elin Yulinah

**Affiliations:** a Animal Health Research & Development, Medion Farma Jaya, Jalan Raya Batujajar 29, Bandung, West Java 40552, Indonesia; b Department of Pharmacology and Clinical Pharmacy, Bandung Institute of Technology (ITB), Indonesia

**Keywords:** AGPs, Phytobiotic, *Andrographis paniculata*, *Origanum vulgare*, Poultry performance, Anticoccidia

## Abstract

The objective of this study was to investigate the effect of phytobiotics combination of *Origanum vulgare* and *Andrographis paniculata* water extracts (FOA) mixed into the feed of broiler and specific-pathogen-free chickens as an alternative to Antibiotics Growth Promoter (AGP). Performance, intestinal bacteria characteristic, and oocysts of *Eimeria* spp. in feces were measured and compared with the AGP-added group. The first experiment in broiler chickens compared FOA, Zinc Bacitracin (ZB, as an AGP group), and negative control. On day 28, FOA group and ZB group showed significantly higher body weight than the control group (*P* < 0.05). The FCR of ZB group was better than FOA group. However, FOA group displayed better microbiota profile than ZB group and negative control, with more *Lactobacillus* spp*. a*nd *Bacillus* spp., and less *Escherichia coli* and *Salmonella* spp. isolated from intestines. The second experiment in specific-pathogen-free chickens showed the anticoccidial effect of FOA addition to reduce the number of oocysts per gram (OPG) from live coccidia vaccine. FOA group and Amprolium group showed OPG reduction (82.53% and 92.02%, respectively) after 7 days of treatment. In conclusion, the combination of *Origanum vulgare* and *Andrographis paniculata* extract can function as an AGP replacement in feed.

## 1 Introduction

The ban on the use of Antibiotics Growth Promoters (AGP) leads to decreasing poultry performance and increasing incidence of pathogenic infections that cause a burden on production cost and economic loss (Cardinal et al., 2019). Zinc bacitracin and Amprolium are commonly used before AGP and coccidiostat were banned (Gadde et al., 2018; Martin et al., 2022). The development of alternatives to AGPs must be carried out to maintain poultry performance and keep production cost-efficient. One approach on developing AGP alternatives is using empirical observational strategies. Probiotics, prebiotics, antimicrobial peptide, polyphenol, and natural extract are several examples of this empirical approach (Brown et al., 2017). Natural extract or phytobiotic feed additives (PFA) have demonstrated favorable impacts on poultry output among potential substitutes that are natural, non-toxic, and residue free. (Gheisar et al., 2015; Yitbarek, 2015; Abudabos et al., 2018). Due to its pharmacological properties, PFA has long been used. It has been hypothesized that herbs, spices, and extracted oils can promote endogenous enzyme secretion, boost antioxidant status, encourage feed intake, and exhibit antimicrobial effects (Lee et al., 2015; Kim et al., 2016; Gheisar & Kim. 2018).

Feed additives with advantageous bioactive components that can protect against microbes and parasites have been developed to improve the performance of broilers. Oregano (*Origanum vulgare*), an aromatic-medicinal plant found in Mediterranean nations, is one potential source of bioactive chemicals acting as a natural growth promoter with strong anticoccidial and antibacterial capabilities (Akrayi et al., 2015; Bozkurt et al., 2016; Pop et al., 2019). It has been demonstrated that oregano and its primary bioactive components (carvacrol and thymol) have synergistic/additive effects such as the antifungal, antiparasitic, antioxidant, positive impact on intestinal microbiota, and improve intestinal cell activity (Zhang et al., 2014; Tzora et al, 2017; Bauer et al, 2019).

Experimentally, *Andrographis paniculata* has antiviral, antimicrobial, antioxidant, immunomodulatory, anti-inflammatory, anti-tumour, chemo preventive, spasmolytic, uterorelaxant, antithrombotic, and antimalarial effect (EMA, 2014). Andrographolide, the main constituent of *A. paniculata* is believed to play important roles in antioxidant, antimicrobial, and antiparasite activity (Dai et al, 2019). In some trials, *A. paniculata* has shown to have potent impacts against *Eimeria* spp*.*, the causative agent of coccidiosis (Setyorini et al, 2016; Indrati & Titisari, 2020) and inhibit pathogenic bacteria growth resulting improve immune status in poultry (Hertamawati et al, 2019).

The potential interactions between different combinations of bioactive ingredients have not been studied in depth. This experiment aims to determine the effect of the dietary use of *A. paniculata* and *O. vulgare* water extracts combination (FOA) on the performance and intestinal microbiota profile as an alternative to AGP addition in feed. Additionally, a preliminary experiment was conducted to see how the FOA affect the oocysts of *Eimeria* spp*.*

## 2 Material and methods

### 2.1 Animals and management

In Experiment I, one-day-old (DOC) Cobb broiler chickens of mixed sex weighing 42–52 gram were used. On day 1, all chickens were vaccinated against Infectious Bronchitis (live), Avian Influenza (killed), and New Castle Disease (live and killed). On day 10, all chickens were vaccinated against Infectious Bursal Disease (live). All chickens were raised in a commercial closed house with a stocking density of 8–10 chickens / m^2^ and husks spread on the floor as pen litter under 24 h lighting. Temperature, humidity, and wind speed in the pen were monitored and controlled through digital panels per the requirements of Cobb 2021. Prior to the experiment, the pen and equipment were disinfected using liquid disinfectant and fumigated with powder formaldehyde.

In Experiment II, 14-day-old male Hy-Line W-36 specific-pathogen-free chickens were raised in battery cages with a Bio-safety level 2 environment. The battery cage was made of stainless steel with the dimension of 45 × 45 × 50 cm and had fecal collection trays beneath the cages. Lighting was provided for 24 h everyday. Feed and drinking water were given individually to each chicken. Prior to the experiment, the pen and equipment were disinfected and passed the sterility test.

### 2.2 Extracts and medicine

The extracts used in this experiment were a commercial 10:1 aqueous extract of *O. vulgare* from Hunan Nutramax, China and a 25% aqueous extract of *A. paniculata* from Phytochemindo Reksa Indonesia. Both powder extracts were formulated into a final product containing 0.5% *O. vulgare* extract and 0.75% *A. paniculata* extract (Optigrin, Medion Farma Jaya, Indonesia). Zinc bacitracin was used as the positive control in the first experiment, while amprolium was used in the second experiment.

### 2.3 Experimental design and dietary treatments

All experiments were conducted according to WAAVP (World Association for the Advancement of Veterinary Parasitology) guidelines for evaluating the efficacy of anticoccidial drugs in chickens and turkeys.

In experiment I chickens were randomly divided into 3 groups each consisting of 250 birds. Each treatment was replicated 5 times containing 50 birds per replicate. FOA group was supplemented with basal diet plus 10 mg of *Origanum vulgare and 15 mg* of *Andrographis paniculata* extract per kg of feed, ZB group was supplemented with basal diet plus 6.3 mg/kg feed of Zinc bacitracin as positive control. Control group was given basal diet without AGP addition as negative control. All groups were kept in the same pen and separated by a partition. Feed supplementation was given ad libitum for 28 days. Concomitant treatments were prohibited except where deemed necessary and would not influence the performance of any product. Body weight (BW) was recorded weekly with 10% samples of the total population according to Cobb standard (2021) while Feed Intake (FI) was recorded daily. Quantification of intestinal microbiota was conducted at the end of the experiment on 5 chickens per group.

Experiment II was conducted to evaluate the anticoccidial activity of FOA compared with Amprolium (AMP). SPF chickens were randomly divided into 3 groups each consisting of 9 birds (3 birds per replicate) according to the sample size in an animal study by Ko & Lim (2021). Fourteen-days old chickens were infected with approximately 35,000 oocysts from the live coccidia vaccine. All chickens showed clinical signs after 6 days of infection. On the next day, FOA group was treated with 10 mg of *Origanum vulgare* extract and 15 mg of *Andrographis paniculata* per kg of feed, AMP group was treated with 10 mg per kg feed of Amprolium, the remaining group was left untreated to serve as control. Oocyst per gram feces (OPG) from each group was quantified 7 days after treatment with FOA or AMP.

The non-medicated commercial feed for both experiments was based on the nutritional value of National Research Council (NRC). Samples were coded and not disclosed to the researchers during the data analysis. No other treatments were given in both experiments.

### 2.4 Poultry performance and intestinal microbiota examination

Average daily weight (ADG) and feed conversion ratio (FCR) were calculated using BW and FI data (*n* = 50). At the end of the experiment, on day 28, five chickens from each group were randomly sacrificed. Duodenum, jejunum, and ileum were separated using a cord on the border of the intestine's part, then all samples were frozen immediately (*n* = 5) (sample size was calculated based on the ANOVA method). For the analysis, the intestine was defrosted and then cut into separate parts of the duodenum, jejunum, and ileum. The content of the intestine parts was collected and mashed. Around 2 grams of intestinal content was suspended in 18 mL of NaCl 0.9% and diluted with 10-fold serial dilution. Every 0.1 mL of the dilution was inoculated into HiChrome Bacillus Agar (HBA) for *Bacillus* spp. quantification, De Man, Rogosa and Sharpe Agar (MRSA) for *Lactobacillus* spp. quantification; and MacConkey Agar (MCA) for *Eschericia coli* quantification. HBA was incubated for 18–24 h, MRSA and MCA for 24–72 h. Incubation temperature was set at 35 – 37°C. Bacteria quantification was performed according to Sajitha et al. (2014), Devi et al. (2013) and Jin LZ et al. (1996).

### 2.5 *.* Coccidial oocyst count

In experiment II, a live coccidia vaccine (Coccivac D, MSD Animal Health, NJ, USA) containing 3.5 × 10^4 oocysts of mixed *Eimeria* spp. was inoculated to each chicken at 14-days old. Before treatment, three chickens were euthanized to see the preliminary anatomical pathology, intestinal score, and OPG. FOA and AMP were given for seven days post-infection (21-days old). Lesion score and OPG were examined on day 0, day 3, day 5 and day 7 of treatment. OPG was calculated using the McMaster technique according to RCV/FAO guidelines. Intestinal lesions was scored based on Johnson & Reid Method (1970), then OPG based on (Jankiewicz et al., 1972)

### 2.6 Statistical analysis

The data were statistically analyzed using SPSS version 18.0. Differences between other groups were tested by the ANOVA-DUNCAN test, with *P* < 0.05 as the significant level.

## 3 Results

### 3.1 Growth performance of broiler fed with dietary additives

Experiment I using broiler chickens was conducted to observe the effect of FOA on performance and microbiota profile compare with ZB as positive control and negative control. The parameter of performance observed in this study are BW, FI, and FCR (Table 1). Groups fed with dietary additives showed superior body weight and FCR throughout the experiment. The body weight and average daily gain of the FOA group and ZB group were not significantly different. Both FOA and ZB groups had lower feed intake than the control group. The FCR of the FOA and ZB group was identical but significantly different from the control group (*P* < 0.05). This indicates that the administration of a herbal combination exhibits performance on equivalent with AGP.

**Table 1:** Effect of feed dietary additives on growth performance in broiler.

Performance parameter	Control	FOA	ZB	SEM
BW (g)				
7 days	185.4 ^a^	197.04 ^b^	195 ^ab^	2.05
14 days	485.8 ^a^	494.12 ^a^	499.36 ^a^	4.67
21 days	983.76 ^a^	1022.72 ^b^	1025.44 ^b^	7.46
28 days	1579.52 ^a^	1663.92 ^b^	1682.84 ^b^	14.61
ADG (g)				
7 days	19.82 ^a^	21.49 ^b^	21.19 ^ab^	0.29
14 days	42.91 ^a^	42.44 ^a^	43.48 ^a^	0.62
21 days	71.14 ^a^	75.51 ^b^	75.15 ^b^	0.64
28 days	85.11 ^a^	91.60 ^ab^	93.91 ^b^	1.4
1 to 28 days	54.75 ^a^	57.76 ^b^	58.44 ^b^	0.52
FI (g/week)				
7 days	180	168	172	
14 days	620.33	547.59	591.75	
21 days	1285.58	1232.47	1217.29	
28 days	2205.94	2150.92	2116.85	
FCR				
7 days	0.97 ^b^	0.85 ^a^	0.88 ^a^	0.01
14 days	1.28 ^c^	1.11 ^a^	1.19 ^b^	0.01
21 days	1.31 ^b^	1.21 ^a^	1.19 ^a^	0.01
28 days	1.40 ^b^	1.29 ^a^	1.26 ^a^	0.01

Different superscripts at the same row are significantly different (*P* < 0.05).

### 3.2 Intestinal microbiota profile of broiler fed with dietary additives

**Fig. 1:**
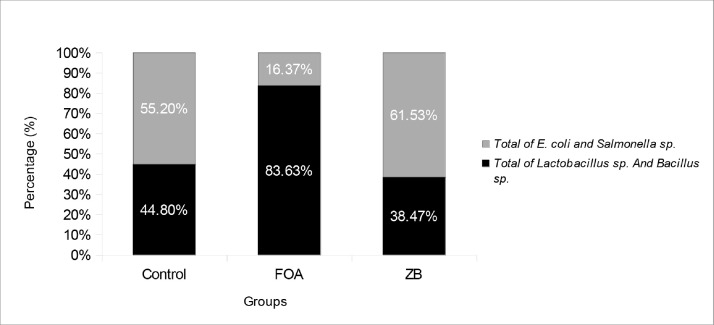
Effect of feed dietary additives on microbiota profile.

This study examined the effects of feed additives on the profile of the intestinal microbiota. The comparison between beneficial bacteria (*Lactobacillus* spp*.* and *Bacillus* spp.) and pathogenic bacteria (*E.coli* and *Salmonella* spp*.*) was displayed in Fig. 1. The percentage of beneficial bacteria in the FOA group was higher than pathogenic bacteria, while it was lower in both ZB and control groups. The ZB group showed the lowest percentage of beneficial bacteria. This data revealed that the use of antibiotics could affect the composition of beneficial bacteria residing in the intestines.

The microbiota profile from each segment of the small intestine can be seen in Table 2. FOA group showed the highest amount of beneficial bacteria in all segments of small intestine (duodenum, jejunum, and ileum) among the other group, while control and ZB group showed higher amount of pathogenic bacteria in all segment of the small intestine.

**Table 2:** Effect of feed dietary additives on intestinal bacteria in broiler chicken intestinal population.

Bacterial Load (Log CFU/g)	Control	FOA	ZB
Total of *Lactobacillus* sp*.* and *Bacillus* sp*.*			
Duodenum	4.61	8.43	4.08
Jejunum	5.87	9.26	5.90
Ileum	8.80	12.56	6.43
Total of *E. coli* and *Salmonella* sp*.*			
Duodenum	7.91	2.12	8.77
Jejunum	8.54	2.28	10.37
Ileum	7.46	1.52	7.11

### 3.3 Anticoccidial activity of SPF chickens fed with dietary additives

The anticoccidial activity was analyzed through OPG quantification. The initial number of OPG in each group was not significantly different as shown in Table 3. After treatments, the OPG count of FOA and AMP groups were reduced significantly (*P* < 0.05) compared to the Control group. At day-7, in FOA and AMP groups were significantly lower than Control group.

**Table 3:** Effect of feed dietary additives on fecal oocyst.

Day of Treatments	Control	FOA	AMP	SEM
Before treatments	28300^a^	15550^a^	10233^a^	3777.02
Day – 3	85400^b^	1883^a^	217^a^	18429.78
Day – 5	22588^b^	1233^a^	33^a^	3864.27
Day – 7	207500^b^	2717^a^	817^a^	42828.52

Different superscripts at the same row are significantly different (*P* < 0.05).

### 3.4 Intestinal lesion of SPF chickens fed with dietary additives

There was no significant difference in intestinal lesion in all groups. Even so, the control group had worsening intestinal lesion day by day, while the FOA and AMP groups were slowly improving in 16 days (Table 4).

## 4 Discussion

In veterinary medicine, antibiotics are frequently used to treat bacterial animal diseases and to protect the health of farm animals (Hockenhull et al., 2017). Due to the low ability of chicken intestine's to absorp many antimicrobials, a huge amount of the drug is excreted in feces unaltered. The widespread use of animal excrement as fertilizer in many nations raises concerns about the negative environmental impacts of antibiotic residues that might contaminate over wide geographical areas (Hao et al., 2014). WHO states that there are currently serious worries regarding the spread of antibiotic resistance genes in bacteria detected in human patients, probably as a result of veterinary usage of antibiotics (WHO, 2014). Additionally, the general public is now demanding reduced use of drugs in livestock production, including ionophores or chemical coccidiostats, as well as antibiotic growth promoters. Due to increased research and exploration of safe and effective solutions to improve performance and decrease coccidiosis, herbal extracts are being utilised (Giannenas et al., 2020; Franz et al., 2020).

Herbal extracts are increasingly used as a safe and effective solution to improve performance and decrease coccidiosis cases. *O. vulgare* and *A. paniculata* have been separately reported to improve performance when added in the feed. However, there were no studies of the use of both extracts combined. The purpose of this study is to determine whether combining *O. vulgare* and *A. paniculata* extract (FOA) as a dietary additive can provide anticoccidial activity comparable to AGP without the need for withdrawal period or the potential adverse effects of anticoccidial drugs.

In the first experiment in broiler, the performance of broiler chickens fed with either FOA or ZB was better than control without dietary additive. The effect of FOA addition was not significantly different to ZB addition. FOA is as effective as ZB (p > 0.05) for optimizing broiler performance. In accordance with previous study by Scocco et al., 2017, feed supplemented with 2 g/kg feed *O. vulgare* aquoeus extract showed the highest body weight at 21 days of treatment compared to vitamin E and control groups. Ri et al., (2017) also stated that use 150 mg/kg of *O.vulgare* powder in the broiler chicks diet significantly increased the body weight and improved FCR as effective as antibiotic virginiamycin. Feed supplemented with 30% dry *A. paniculata* leaves could increase ADG and reduce FCR (Hidanah et al., 2020). Our study showed that lower dosage of both extracts, 15 mg of *A. paniculata* and 10 mg of *O. vulgare* extract per kg of feed, when used in combination can provide similar results.

**Table 4:** The average of intestinal (duodenum, jejunum, ileum, caecum) lesion score.

Days of Treatments	Treatments			SEM
	Control	FOA	AMP	
Before treatments	1.25^a^	1.25^a^	1.25^a^	0
After treatments				
Day – 2	1.83^a^	1.42^a^	1.58^a^	0.09
Day – 10	2.00^a^	2.08^a^	1.92^a^	0.09
Day – 16	2.08^a^	1.75^a^	1.75^a^	0.13

Different superscripts at the same row are significantly different (*P* < 0.05).

Lesion Scoring: 0 = no lesions; 1 = mild lesions; 2 = moderate lesions; 3 = severe lesions;

4 = extremely severe lesions and death (Johnson & Reid Method,1970)

The dietary addition of FOA in broiler can shift the intestinal microbiota profile towards beneficial bacteria. FOA group showed highest amount of beneficial bacteria in all segment of small intestine (duodenum, jejunum, and ileum) among the other groups. As the number of pathogenic bacteria decreases, beneficial bacteria are able to increase. Previous studies using *O. vulgare* aqueous extract to reduce *E.coli* and the other pathogen bacteria in poultry intestines. Hidanah et al. (2020) also reported that feed supplemented with aqueous *A. paniculata* extract could reduce *E.coli* bacterial infection. This is in accordance with the statement of Aruwa et al. (2021), that beneficial bacteria such as *Lactobacillus* spp. was predominant in small intestine, especially in the ileum. One interesting result in this study is that ZB addition results in the least amount of beneficial bacteria. It confirms with the study by Johnson et al. (2015) that antibiotics can have an adverse effect on gut communities by reducing the abundance and diversity of commensal microbes. Higher number of pathogenic bacteria could be caused by ZB resistancy in *E. coli* (Boulianne et al., 2016).

It is well known that the bacterial cell wall is the primary target for the antibacterial effects of phenols. The aqueous extract of *O. vulgare* contained a high concentration of phenolic compounds and was a powerful antioxidant (Teixeira et al., 2013). The mechanism of *O. vulgare* was likely due to its ability to penetrate cell membranes and disrupt their integrity. Gholami et al. (2022) stated that if bacteria cells come into contact with oregano, fluids would leak out from the bacteria due to one of its active ingredients, phenolic compound (carvacrol or thymol). The internal pH turned acidic, impairing metabolism and replication. These finding was consistent with electron microscope observations that revealed the damage to bacteria cell membranes exposed to oregano (Gholami et al., 2022). *A. paniculata* primary bioactive compound, andrographolide, had been reported in several studies to have antibacterial activity. Andrographolide worked to inhibit bacterial growth by inhibiting DNA synthesis, almost as effectively as the fluoroquinolone class of antibiotics (Banerjee et al., 2017). *O. vulgare* and *A. paniculata* exhibited antimicrobial action that targets two different bacterial pathways to synergistically reduce pathogenic bacteria, causing a balance of microbiota in the intestines of chicken. This balance of microbiota resulted in more optimal nutritional absorption in the intestine, hence improves poultry performance (Gholami et al., 2022).

In this experiment, treatment with FOA can control the number of *Eimeria* spp*.* as effective as AMP (*P* > 0.05). Studies conducted by Setyorini (2016) and Indrati & Titisari (2020), showed that *A. paniculata* extract supplemented in broiler feed could reduce the number of *Eimeria tenella* oocysts*.* According to Bozkurt et al. (2016), dietary oregano essential oil supported the intestinal absorptive capacity and antioxidant defense system during *Eimeria* infection; however, it displayed minimal direct activity on the reproductive capacity of *Eimeria*. The commercial herbal formula of *O. vulgare, Satureja horetensis*, and *Chelidonium majus* could reduce the cecal lesions but had no efficacy on *Eimeria* spp. infection shown by anticoccidial index (Pop et al., 2019). This study proves that the combination of *O. vulgare* and *A. paniculata* has anticoccidial activity to reduce oocyst in FOA-treated groups. Although the mechanism of action has not been clear, a reasonable explanation for this anticoccidial activity is the hydrophobic character and low molecular weight of the main phenolic compounds present in those that allow them to disintegrate outer cell membrane (Jitviriyanon, 2016). Furthermore, the high lipid solubility of *O. vulgare* and *A. paniculata* is likely to permit rapid diffusion through parasite and host cell membranes. Other possible mechanism is the interference of calcium-mediated signaling necessary for invasion by *E. Tenella* sporozoites (Jitviriyanon, 2016). The content of andrographolid in *A. paniculata* acts to increase immunity. Andrographolide found in *A. paniculata* can improve immune function. Interferon produced by lymphocytes increased the response of phagocytic activity by macrophage cells to inhibit the replication of *E. tenella* (Dai et al., 2019).

Decreased lession score in both FOA and AMP-treated groups is observed in this study. *O. vulgare* and *A. paniculata* have anti-inflammatory activity that can inhibit inflammatory mediators caused by bacterial and parasitic infections such as prostaglandins, interleukins (IL), interferons (IFN) and tumor necrosis factor (TNF), etc (Gholami et al., 2022; Dai et al., 2019).

From this experiments, there were no adverse effects reported in all treatment groups. More extensive large scale studies with *in-vitro* and *in-vivo* challenge are required to confirm this.

## 5 Conclusion

The supplementation of 10 mg of *Origanum vulgare* and 15 mg of *Andrographis paniculata* extract (FOA) can increase broiler performance and reduce pathogenic intestinal bacteria as good as Zinc Bacitracin. FOA reduces pathogenic intestinal bacteria better than Zinc Bacitracin. In addition, FOA has anticoccidial activity to reduce oocyst per gram feces of *Eimeria* spp*.* as effective as Amprolium. Based on this study, FOA can be used as an alternative to antibiotic growth promoters. Further research is required to identify and quantify the chemical content of both extracts that improve the poultry performance.

## Declaration of Competing Interest

The authors declare that they have no known competing financial interests or personal relationships that could have appeared to influence the work reported in this paper.
